# Characterization and differential expression of microRNAs elicited by sulfur deprivation in *Chlamydomonas reinhardtii*

**DOI:** 10.1186/1471-2164-13-108

**Published:** 2012-03-22

**Authors:** Longfei Shu, Zhangli Hu

**Affiliations:** 1 Dept. of Aquatic Ecology, Eawag, Switzerland; 2 Institute of Integrative Biology, ETH-Zurich, Switzerland; 3 Shenzhen Key Laboratory of Marine Bioresource and Eco-environmental Science, College of Life Sciences, Shenzhen University, Shenzhen 518060, Peoples Republic of China

## Abstract

**Background:**

microRNAs (miRNAs) have been found to play an essential role in the modulation of numerous biological processes in eukaryotes. *Chlamydomonas reinhardtii *is an ideal model organism for the study of many metabolic processes including responses to sulfur-deprivation. We used a deep sequencing platform to extensively profile and identify changes in the miRNAs expression that occurred under sulfur-replete and sulfur-deprived conditions. The aim of our research was to characterize the differential expression of *Chlamydomonas *miRNAs under sulfur-deprived conditions, and subsequently, the target genes of miRNA involved in sulfur-deprivation were further predicted and analyzed.

**Results:**

By using high-throughput sequencing, we characterized the microRNA transcriptomes under sulphur-replete and sulfur-deprived conditions in *Chlamydomonas reinhardtii*. We predicted a total of 310 miRNAs which included 85 known miRNAs and 225 novel miRNAs. 13 miRNAs were the specific to the sulfur-deprived conditions. 47 miRNAs showed significantly differential expressions responding to sulfur-deprivation, and most were up-regulated in the small RNA libraries with sulfur-deprivation. Using a web-based integrated system (Web MicroRNAs Designer 3) and combing the former information from a transcriptome of *Chlamydomonas reinhardtii*, 22 miRNAs and their targets involved in metabolism regulation with sulfur-deprivation were verified.

**Conclusions:**

Our results indicate that sulfur-deprivation may have a significant influence on small RNA expression patterns, and the differential expressions of miRNAs and interactions between miRNA and its targets might further reveal the molecular mechanism responding to sulfur-deprivation in *Chlamydomonas reinhardtii*.

## Background

Sulfur is an essential trace element for all organisms, and is widely used in biochemical processes. Many enzymes and antioxidant molecules such as glutathione contains sulfur. Organically bonded sulfur is a component of all proteins, in the amino acids cysteine and methionine. Generally, sulfate is the most stable form of sulfur. The available pools of sulfate can vary significantly as environmental conditions change. Most organisms have a limited capacity to store sulfur, and thus require different strategies to optimize sulfur use for survival. The ability of microbes to acclimate to periods of nutrient insufficiency is essential to their survival in the natural environment [1]. The unicellular green alga *Chlamydomonas reinhardtii *is an ideal model organism for the study of many metabolic processes including response to sulfur-deprivation. Sulfur-deprived *Chlamydomonas *cells have been used for microarray-based RNA abundance studies [2,3], RNA-seq analysis [4], determination of metabolite profiles [5], and sustained production of H_2 _[6-8]. *Chlamydomonas reinhardtii *exhibits several responses to sulfur deprivation, including changes of the photosynthetic apparatus, the synthesis of enzymes, cell wall structure, SO_4_^2- ^transport activity, and cell size [4].

Recently, there has been considerable interest in understanding the impacts of sulfur deprivation on miRNA. miRNAs were first found in *Caenorhabditis elegans *through forward genetic screens of the *lin-4 *and *let-7 *mutants [9,10]. Since then, genetic studies on various organisms have revealed that miRNAs are universally present and are key components of various gene regulatory pathways in eukaryotes. *Chlamydomonas *miRNAs were discovered independently by two groups [11,12], showing that miRNAs exist not only in multicellular systems but also in unicellular eukaryotes. Experimental approaches and bioinformatics-assisted screening have identified approximately 85 *Chlamydomonas reinhardtii *miRNAs, which are listed in the miRBase version15.0 http://microrna.sanger.ac.uk/s-equences/index.shtml. To investigate the role of *Chlamydomonas reinhardtii *miRNAs involved in sulfur deprivation, we used a deep sequencing platform to extensively profile and identify changes in the miRNAs expression that occur during sulfur-replete and sulfur-deprived conditions.

## Results

### The small RNA profile of *Chlamydomonas reinhardtii *in both sulfur-replete and sulfur-deprived conditions

**Figure 1:**
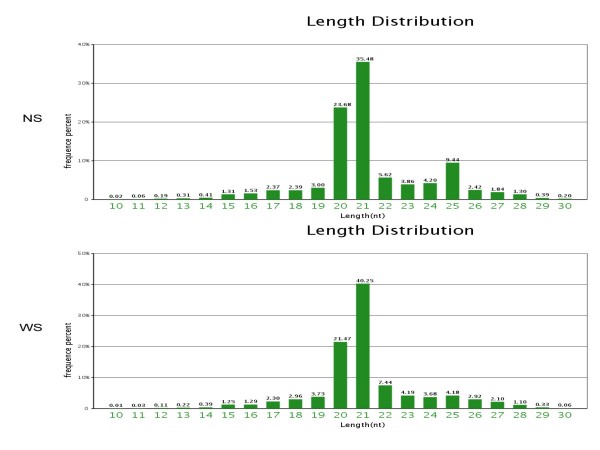
**The size distribution of small RNAs in both + S (NS) and -S (WS) libraries**.

**Figure 2:**
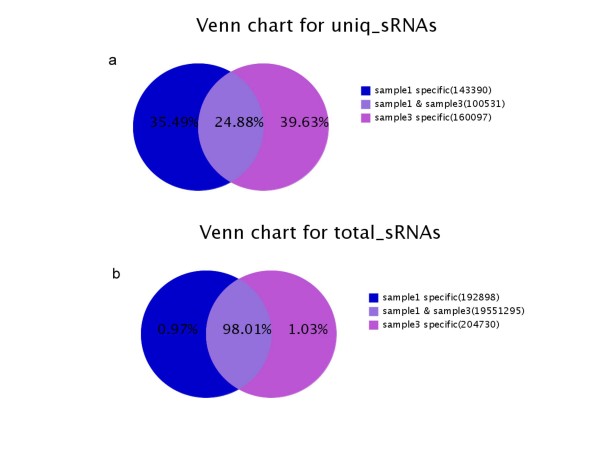
**Summary of the common and specific tags of small RNAs in + S (sample 1) and -S (sample 3) libraries, including the summary of unique tags (a) and total tags (b)**. (a) sample1 specific: number of unique sample 1 (+S) specific sRNAs and the percentage; sample3 specific: number of unique sample 3 (-S) specific sRNAs and the percentage; sample1 & sample3: number of unique common sRNAs between two samples and percentage. (b) sample1 specific: number of total sample1 (+S) specific sRNAs and the percentage; sample3 specific: number of total sample3 (-S) specific sRNAs and the percentage; sample1 & sample3: number of total common sRNAs between two samples and percentage.

The equal numbers of algal cells were resuspended under continuous illumination for up to 72 h in TAP (with sulfate 40.55 mg/L) or TAP-S (the sulfate concentration was less than 0.42 mg/L). Two small RNA libraries were constructed using the algal cells under sulphur-replete (+S library) and with sulfur-deprived conditions (-S library) respectively. Sequencing of the *Chlamydomonas reinhardtii *small RNA libraries was performed with Solexa high-throughput sequencing. We obtained 11,080,539 reads from the sulfur-deprived (-S) library and 11,284,767 reads from the sulfur-replete (+S) library. After discarding low quality and shorter than 18 (nt) sequences, 9,918,931(-S library) and 10,029,992 (+S library) clean reads ranging from 18 to 30 nt were collected (Table 1). The majority of the small RNA sequences obtained from the -S and + S libraries were 20-25 nt in size, which was the typical size range for Dicer derived products (Figure 1). Comparing the common small RNAs sequences between -S and + S libraries, although the total small RNAs sequences in both libraries reached 98.01%, the common categories of unique small RNAs were only 24.88%. It indicates that sulfur-deprivation led to a significant influence on the small RNA expression patterns in *Chlamydomonas reinhardtii *(Figure 2).

**Table 1:** Statistics of small RNA sequences from *Chlamydomonas reinhardtii *+ S and -S libraries

	Sequences generated	%
**+S library**		
Total reads	11284767	
High quality	10762852	100%
Adaptor3 null	3304	0.03%
Insert null	2815	0.03%
Adaptor5 contaminants	63608	0.59%
Smaller than 18 nt	663116	6.16%
PolyA	17	0.00%
Clean reads	10029992	93.19%
**-S library**		
Total reads	11080539	
High quality	10572756	100%
Adaptor3_null	3499	0.03%
Insert null	3575	0.03%
Adaptor5 contaminants	58280	0.55%
Smaller than 18 nt	588466	5.57%
PolyA	6	0.00%
Clean reads	9918931	93.82%

**Total reads**: total sequenced reads; **High quality**: number of high quality reads with no N, no more than 4 bases whose quality score is lower than 10 and no more than 6 bases whose quality is lower than 13; **Adaptor3 null**: number of reads with no 3' adaptor; **Insert null**: number of reads with no insertion; **Adaptor5 contaminants**: number of 5' contaminants; **Smaller than 18 nt**: number of reads less than 18 nt. Generally, small RNA tags are between 18 ~ 30 nt long. So too short tags should be removed from data for future analysis; **polyA**: number of reads wih polyA; **Clean reads**: number of clean reads after adaptors and contaminants are removed which are used in the following analysis.

**Figure 3:**
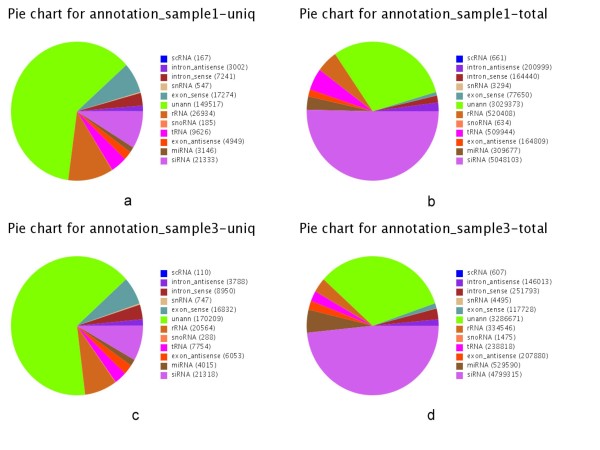
**Small RNA annotation of + S (a, b) and -S (c, d) libraries**. Charts a and c show the numbers of unique sRNA tags matched to all categories of RNA; charts b and d show the number of total sRNA tags matched to all categories of RNA.

The 20-24 nt sequences from the + S and -S libraries were aligned to the draft *Chlamydomonas reinhardtii *genome using SOAP [13]. A total of 4,598,243 (+S) and 6,039,480 (-S) sequences were found to match the genome perfectly (see Additional file 1: Figure S1). These small RNAs were used for further analysis. With the alignment to Genbank, Rfam, Exon and Intron, the composition of small RNAs were annotated as siRNA, miRNA, snRNA, snoRNA etc. (Figure 3). Among all the categories of small RNAs, siRNAs expression (small interfering RNA) did not change on sulfur deprivation. However, the microRNAs expression showed more significant up-regulation in response to sulfur-deprivation (Table 2).

**Table 2:** Composition of the small RNAs in the + S and -S libraries

	Unique sRNA (percentage values)		Total sRNA (percentage values)	
	**+S**	**-S**	**+S**	**-S**
Total	243921	260628	10029992	9918931
Exon antisense	4949 (2.02)	6053 (2.3)	164809 (1.6)	207880 (2.09)
Exon sense	17274 (7.08)	16832 (6.4)	77650 (0.7)	117728 (1.1)
Intron antisense	3002 (1.2)	3788 (1.46)	200999 (2.0)	146013 (1.4)
Intron sense	7241 (2.9)	8950 (3.4)	164440 (1.63)	251793 (2.5)
miRNA	3146 (1.2)	4015 (1.5)	309677 (3.0)	529590 (5.3)
rRNA	26934 (11.0)	20564 (7.8)	520408 (5.1)	334546 (3.3)
scRNA	167 (0.06)	110 (0.04)	661 (0.00)	607 (0.00)
siRNA	21333 (8.7)	21318 (8.1)	5048103 (50.3)	4799315 (48.3)
snRNA	547 (0.2)	747 (0.2)	3294 (0.032)	4495(0.04)
snoRNA	185 (0.07)	288 (0.11)	634 (0.00)	1475 (0.01)
tRNA	9626 (3.4)	7754 (2.9)	509944 (5.08)	238818 (2.0)
unann	149517 (61.2)	170209 (65.0)	3029373 (30.2)	3286671 (33.0)

### Identifying novel potential miRNAs in *Chlamydomonas reinhardtii*

**Figure 4:**
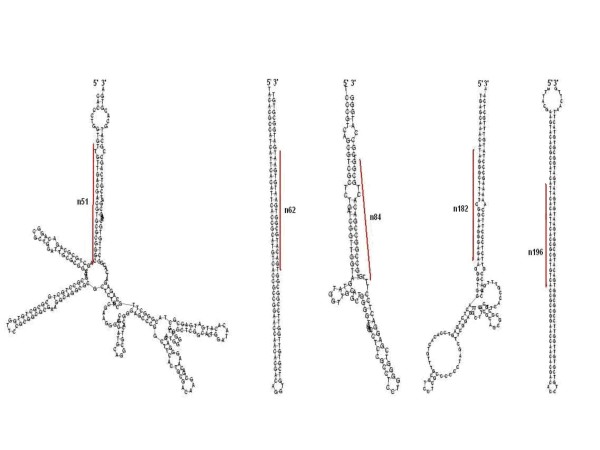
**The hairpin secondary structures of 5 predicted miRNA precursors (n51, n62, n84, n182, n196)**. The positions of mature miRNAs are highlighted in red.

To date, miRBase had a collection of 85 *Chlamydomonas *miRNA. The characteristic hairpin structure of miRNA precursor can be used to predict novel miRNA. We used the predictive software Mireap to predict novel miRNAs by exploring their secondary structures, and the minimum free energy of the unannotated small RNA tags which could be mapped to the genome. We predicted 225 novel miRNAs by Solexa sequencing and Mireap predictive software in *Chlamydomonas reinhardtii*. Some of the novel potential miRNAs (n51, n62, n84, n182, n196, Figure 4) have more than ten thousand reads but were not detected by former research [11,12]. These results showed that high-throughput sequencing of small RNAs is also an ideal strategy to analyze small RNAs profiles and identify novel potential miRNAs in *Chlamydomonas reinhardtii*.

### Differential expression of *Chlamydomonas reinhardtii *miRNAs in sulfur-deprived and sulphur-replete conditions

In order to detect the effect of sulfur-deprivation on Chlamydomonas reinhardtii miRNAs, the expression abundance of 310 candidate miRNAs (minimum short RNA sequence depth above 1) in both -S and + S libraries was examined. Overall, approximately 15% (47 miRNA) of miRNAs showed significant changes in expression (fold changes > 2) upon sulfur-deprivation. Among these miRNA with altered expression, 24 known miRNAs and 23 predicted miRNAs were up-regulated with 13 miRNAs being -S library specific (Tables 3, 4).

**Table 3:** Known *Chlamydomonas reinhardtii *miRNAs that are responsive to sulfur-deprivation

pairwise	miR-name	+S-std	-S-std	fold-change (log2 -S/+S)	p-value	sig-lable
+S/-S	miR1144a.1	2.7916	6.8556	1.29619070	3.00277572489945e-05	**

+S/-S	miR1144b	67.7967	174.2123	1.36155953	**4.25092513184362e-107**	**

+S/-S	miR1147.1	645.5638	2759.7732	2.09591812	0	**

+S/-S	miR1148.2	5.2842	13.2071	1.32155674	3.68981982177614e-09	**

+S/-S	miR1149.1	0.1994	1.4114	2.82338960	0.00218223161858286	**

+S/-S	miR1149.2	35.3938	81.9645	1.21150253	4.19876393573889e-43	**

+S/-S	miR1150.3	0.3988	1.2098	1.60103125	0.0464712840858496	*

+S/-S	miR1153.1	1051.8453	2991.5522	1.50797170	0	**

+S/-S	miR1153.2*	44.8654	96.4822	1.10465953	9.53780690450724e-44	**

+S/-S	miR1155	18.9432	63.0108	1.73391907	3.9298538924937e-56	**

+S/-S	miR1156.1	31.6052	729.5141	4.52870201	0	**

+S/-S	miR1156.2	37.1885	302.6536	3.02473905	0	**

+S/-S	miR1158	1.5952	4.5368	1.50793775	0.000148301262915249	**

+S/-S	miR1159.2	0.7976	2.1172	1.40842024	0.0148368997880162	*

+S/-S	miR1160.2	0.6979	36.3951	5.70458009	4.07725858511646e-98	**

+S/-S	miR1160.3	0.0997	9.1744	6.52387649	1.14199975919422e-26	**

+S/-S	miR1164	1.5952	4.0327	1.33800877	0.00109191543677247	**

+S/-S	miR1166.1	0.6979	2.7221	1.96362783	0.000450528886605393	**

+S/-S	miR1172.1	232.3033	921.3694	1.98777004	0	**

+S/-S	miR1172.2	308.0760	1139.3365	1.88683570	0	**

+S/-S	miR906-3p	11.8644	42.5449	1.84234706	1.13973265129334e-41	**

+S/-S	miR909.1	0.3988	1.4114	1.82338960	0.0180099225615881	*

+S/-S	miR910	260.9175	924.7972	1.82554332	0	**

+S/-S	miR912	2830.5107	6891.9725	1.28385457	0	**

pairwise: pair of samples in differentially expression analysis; miR-name: miRNA name; -std: normalized expression level of miRNA in a sample; fold change (log2 -S/+S): fold change of miRNAs in the pair of samples; p-value: p value which reflects the significance of miRNA differential expression between samples. Less p value shows more significance of difference of miRNA between samples; sig-label: significance label, **: fold change (log2) > 1 or fold change (log2) < -1, and p value < 0.01. *: fold change (log2) > 1 or fold change (log2) < -1, and 0.01 < = p value < 0.05

**Table 4:** Predicted *Chlamydomonas reinhardtii *miRNAs that are responsive to sulfur-deprivation

Name	Sequence (5'-3')	L (nt)	Location in the geonome	MFE (kcal mol^-1^)	Reads +S/-S
n006	UCCAGCUGGGCGGCCGUCUCC	21	chromosome_10:4733426:4733510:-	-38.6	1004/2798
n015	UUCUACCCAAGAGGCUGUGUA	21	chromosome_12:2479175:2479337:+	-93.9	84/264
n030	UCAAAGCUAGGAGCCAUGAAG	21	chromosome_14:139326:139506:+	-129.4	1347/4625
n046	UGUUCGGAGAUCCUUGUGCAUG	22	chromosome_16:3072001:3072107:-	-101.1	117/373
n051	UUGUUGACGACGUGCGCGGGC	21	chromosome_17:204043:204314:-	-129.8	41137/86593
n052	UGACACAUGGAACAACACAAC	21	chromosome_1:3554627:3554814:+	-150.9	287/505
n062	UGACAUGCGGUGAAUGUGAAU	21	chromosome_3:5564702:5564806:+	-105.4	15185/49088
n077	UCAUGAAGCGGAUACUGUGAA	21	chromosome_7:743887:744064:-	-103.91	1160/61
n083	UGGGCCUGUUGUGCACGUUCC	21	scaffold_28:208520:208678:-	-115.9	104/662
n084	UUGUGCCGGCCGACACUGCGG	21	scaffold_33:45408:45519:+	-49.9	77092/55878
n152	UGUACGGCGACCUGCAAAUGG	21	chromosome_10:5980485:5980652:+	-115.7	0/215
n168	AGGAUGACCGUCAUGAUUGCG	21	chromosome_13:4630466:4630659:-	-167	0/4628
n169	GUCAUUAAGACCGUCGGCAAU	21	chromosome_14:1815533:1815835:+	-206.26	0/170
n182	UAGGGCUUUUCGGAAGGGAGA	21	chromosome_17:4302900:4303054:+	-83.2	0/20463
n196	AUUCACAUUCACCGCAUGUCA	21	chromosome_3:5564693:5564816:+	-112	0/16257
n197	UCCUCCUCCUUGACGUCGGCG	21	chromosome_3:7309395:7309503:+	-50.1	0/321
n198	AGCAGCUUCCCACUCCCACGACC	23	chromosome_3:2104211:2104422:-	-66.5	0/415
n200	UGCGCAGCGGCAUCAUCUGGA	21	chromosome_4:2994696:2994894:-	-157.7	0/367
n207	UAGCAGUCUGAACCAAAGUCG	21	chromosome_7:2564607:2564734:+	-92.1	0/931
n209	UAUGGGCAGUUGUACUAAAUC	21	chromosome_7:4354369:4354571:+	-136.8	0/564
n212	UGGGCCUCACGGCGGCGGACC	21	chromosome_7:5449979:5450131:+	-140.8	0/270
n214	AGGGCCAACAGCUUUGACCGG	21	chromosome_7:5670376:5670487:+	-103.9	0/557
n222	AAUGCCAGCAGCUCCACGCCC	21	scaffold_18:257:618:+	-158.6	0/4384

**Name**: the name of predicted *Chlamydomonas reinhardtii *miRNAs that are responsive to sulfur-deprivation; **Sequence**: sequence cloned in small RNA libraries; **L**, the length of miRNA; **MFE**: the adjusted minimum free energy (MFE) representing the MFE of 100 nucleotides; **Reads**: the effect of sulfur-deprivation on miRNA expression, +S/-S, normalized sequencing frequencies in the +S and -S library.

**Figure 5:**
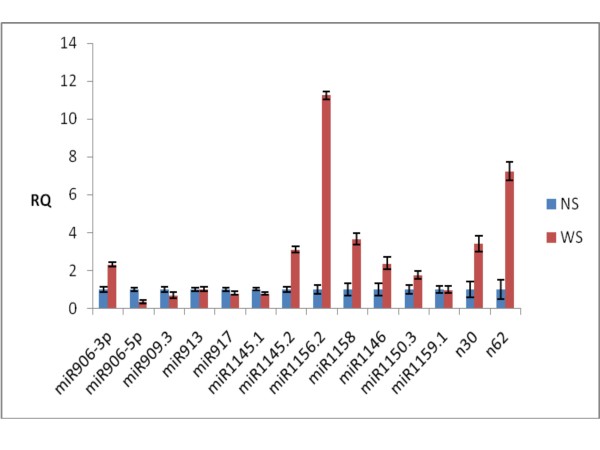
**quantitative RT-PCR analysis of fold changes for the expression of fourteen miRNAs between + S(NS) and -S(WS)**.

To confirm the expression changes of *Chlamydomonas *miRNAs and their response to sulfur-deprived stress, we used quantitative RT-PCR analysis to validate the results of the high throughput sequencing. Fourteen miRNAs, which included 12 known miRNAs and 2 novel predicted miRNAs, were selected at random. The quantitative RT-PCR experimental results of 11 miRNAs matched these of high throughput sequencing data (Figure 5 and Tables 3, 4). However, 3 of the chosen miRNAs did not show the matched results. We deduce that this was likely due to the low quality of primers [14] or low abundance of miRNAs, and further research is needed for this problem.

### Target predictions of *Chlamydomonas reinhardtii *miRNAs involved sulfur-deprivation

The target genes of Chlamydomonas. reinhardtii miRNAs were predicted by a web-base integrated system, Web MicroRNAs Designer 3 http://wmd3.weigelworld.org/cgi-bin/webapp.cgi. The 47 miRNA, which showed differential expressions (fold changes > 2) to sulfur-deprivation were screened for target predictions. We divided the predicted target genes into different group according to their putative functions including photosynthesis, carbon metabolism, lipid metabolism and other processes. We analyzed the differential expression correlation between the miRNA and the target genes [3,4] in response to sulfur-deprivation; consequently, we confirmed 17 miRNAs and the target genes involved in response to sulfur-deprivation in *Chlamydomonas reinhardtii *(Table 5).

**Table 5:** General overview of *Chlamydomonas reinhardtii *miRNAs and their functional targets responded to sulfur-deprivation

Potential Role	miRNAs ID	Expression of miRNA	Functional Target Gene	**Expression of gene **[3,4]
**Lipids metabolism**	miR914	Up	Lipoxygenase	Down
	n196	-S only	SC5D, C-5 sterol desaturase	Down
	n214	-S only	Glycerol-3-phosphate dehydrogenase	Unchanged
	n222	-S only	*LPB1*, lowphosphate bleaching protein	Up
**Proteolisis and peptidolisis**	miR906.3p	Up	26S proteasome regulatory subunit	Unchanged
	miR1147.1	Up	COP signalosome subunit 5	Down
	n198	-S only	Peptidase M14, carboxypeptidase A	Unchanged
	n222	-S only	Peptidase M14, carboxypeptidase A	Unchanged
**Photosynthesis**	miR1150.3	Up	Cytochrome P450 CYP3/CYP5/CYP6/CYP9 subfamilies	Down
	miR1166.1	Up	Phosphatidylglycerophosphate synthase	Down
	miR909.1	Up	pheophorbide a oxygenase	Unchanged
**Carbon metabolism**	miR1158	Up	6-phosphogluconate dehydrogenase, decarboxylating	Up
	n222	-S only	NADP malic enzyme	Down
**Transporters and translocons**	n034	-S only	PTA3, proton/phosphate symporter	Down
	n197	+S only	amino acid transporter	Up
**DNA binding, RNA binding, transcription and translation processes**	miR1166.1	Up	ribosome biogenesis pescadillo-like protein	Down
	n197	+S only	PRPL1, plastid ribosomal protein	Down
**Purine and pyrimidine metabolism**	miR1147.1	Up	Adenylate/guanylate kinase	Down
**Inositol metabolism**	miR1150.3	Up	phosphatidylinositol 3-kinase-related protein kinase	Unchanged
**Amino acid metabolism**	miR1156.2	Up	N-acetyltransferase	Down
**Kinases and phosphatases**	n005	Up	Serine/threonine protein kinase	Up
**Redox processes**	n030	Up	cytochrome P450, CYP85 clan	Unchanged

**Potential role**: potential role of target genes in metabolism; **miRNAs ID**: name of miRNA; **expression of miRNA**: the expression level of *C. reinhardtii *miRNAs after sulfur-deprivation; **functional target gene**: possible functional target genes by target gene prediction; **expression of gene**: the expression of target genes reported by published references

## Discussion

### The reliability of the method to identify *Chlamydomonas *miRNA

miRNAs are a group of small non-coding RNAs that play an important role in various developmental and stress response processes through negative control of gene expression [15]. miRNA identification had been reported in *Chlamydomonas reinhardtii *[11,12], but only 85 miRNAs were annotated and collected to miRBase in previous studies. In this study we characterized a total of 310 *Chlamydomonas reinhardtii *miRNAs which included the 85 known miRNAs and 225 novel miRNAs by using Solexa sequencing technology with prediction software Mireap. We found that most known miRNAs were abundant, and that the 225 novel miRNAs were less abundant or specific to the miRNA of -S library. It further confirmed Solexa sequencing technology as a high-throughput sequencing system, which was able to produce highly accurate, reproducible and quantitative readouts of small RNAs [16,17].

### The *Chlamydomonas *miRNAs expression patterns for responding to sulfur-deprivation

The unicellular green alga *Chlamydomonas reinhardtii *has been used as a model organism for studying the responses of eukaryotes to sulfur-deprivation [4]. The completion of *Chlamydomonas reinhardtii *genome sequencing and substantially expressed sequence tag (EST) project has greatly increased its utility as a model system [18]. Although some studies have been carried out on *Chlamydomonas *miRNAs regulation [11,12], this is the first report on the miRNAs which responded to sulphur-deprivation stress in *Chlamydomonas reinhardtii*. By comparing the expression levels of two miRNAs libraries(+S library and -S library), we found that approximately 15% of miRNAs expressions showed evident changes (fold changes > 2) with sulfur deprivation (Tables 3, 4). These results further indicate that sulfur-responsive processes are complicated and need a lot of miRNAs to participate in the regulation of gene expression at both transcriptional and post-transcriptional levels [3,4]. Interestingly, although most miRNA which were reported by several groups [10,11] were found in this study, we found that no *Chlamydomonas *miRNAs have identifiable orthologs with miR395s. miR395s play a very important roles in the sulfur-deprived response in higher plants [19,20] where miR395 regulates sulfate distribution and metabolism in the plant cells. Further, miR395 targets a low-affinity sulfate transporter AST68 (At5g10180) and 3 enzymes in the sulfate assimilation pathway (i.e., ATP sulfurylases [(APS1: At3g22890), (APS3: At4g14680) and (APS4: At5g43780)] [19,20]. The highly conserved miR395 and the presence of multiple copies of miR395 in diverse plant species is necessary for sulfur homeostasis. The lack of a universally conserved miR395s family in *Chlamydomonas reinhardtii *suggests that green algae miRNAs may have a different pathway for responding to sulfur-deprivation than higher plants.

### Target genes of *Chlamydomonas *miRNAs involved in sulfur-deprivation

miRNAs negatively regulated their targets by cleavage-induced degradation, and the effects of miRNAs over expression were thus often reflected in decreased mRNA levels of the target gene [21]. Although we found the differential expression of multitudinous *Chlamydomonas *miRNA between sulfur-replete and sulfur-deprived conditions, it is difficult to confirm their target genes because of the complex mechanism of interaction between miRNAs and their target transcripts was not determined. Zhao *et al *(2009) selected the precursor of miRNA cre-MIR1162 as backbone to design amiRNAs silencing the MAA7 and RBCS2 genes [22]. Attila *et al *(2009) selected the precursor of miRNA cre-MIR 1157 as a backbone to efficiently produce amiRNAs targeting COX90, PSY and DCL1 genes [23]. James et al (2010) also used amiRNAs silencing HydA1, HydA2, and Hyd3 genes [24]. This far we have not found the specific bioinformatics tools to predict *Chlamydomonas *miRNAs targets, so we chose the web-base integrated system (Web MicroRNAs Designer 3) to predict *Chlamydomonas *miRNAs involved in sulfur-deprivation. The predicted results contained false positive rates similar to other reports on miRNA target prediction [14], so it was necessary to verify these predicted targets. However, it was more difficult to screen the miRNAs targets without the 3' UTR sequence database and abundance information of mRNA expression for response to sulfur-deprivation because most miRNA targeted the 3' UTR of mRNA and expression abundances showed the negative correlative between miRNA and their target mRNA. Fortunately, the *Chlamydomonas reinhardtii *trancriptome was characterized from sulfur-replete and sulfur-depleted conditions [3,4], which provided very important information to analyze the interaction between miRNA and its target mRNA. Theoretically, with the sulfur-deprivation stress, the amount of related mRNAs expression will increased, the transcription levels of its target genes should be decreased by miRNA-mediated gene silencing. In fact, we found that expression abundances of most miRNAs showed a negative correlation with their levels of target mRNA under sulfur-deprivation among 22 miRNAs which regulated the metabolic activities (Table 5). Interestingly, the four target genes which encoded low phosphate bleaching protein (LPB), 6-phosphogluconate dehydrogenase (6-PGDH), decarboxylating amino acid transporter and serine/threonine protein kinase were up-regulated while the expressions of the their miRNA including n222, miR1158, n197 and n005 were up. LPB is important for acclimation of *Chlamydomonas reinhardtii *to phosphorus and sulfur deprivation [25]. 6-PGDH is the first enzyme for pentose phosphate pathway (PPP), which is common for plant responses to abiotic stresses, and serine/threonine protein kinase is required for acclimation of the alga to sulfur deprivation [26]. These four genes are important for *Chlamydomonas reinhardtii *to survive under sulfur-deprivation, so their miRNA should be down regulated when the algal cell is stressed by sulfur deprivation. However, our results from high-throughput sequencing demonstrate that their regulated miRNAs are increased with sulfur deprivation. These results may indicate that the expression of the 4 genes are regulated by multiple factor, the miRNA regulation may not be the major force. Further experiments are needed to verify this hypothesis.

We were particularly interested in the relative miRNA and its targets for hydrogen bioproduction. The differential expression profile of *Chlamydomonas *miRNAs led to massive changes in gene expression and metabolism which was closely associated with H_2 _photo-production. Several miRNAs targeted to genes involved photosynthesis created an anaerobic environment and induced the activity of hydrogenase. The target gene of miR1166.1 encoded the PG phosphate synthase protein. PG is synthesized from cytidyldiphosphate (CDP)-diacylglycerol and glycerol-3-phosphate by the catalytic action of PG-phosphate synthase. Being the only phospholipid in cyanobacteria, PG was required for the accumulation of chlorophyll-protein complexes in the thylakoid membrane and for the normal functioning of photosystem II (PSII) [27]. In the absence of O_2_, in order to generate ATP, green algae resorted to anaerobic photosynthetic metabolism by miRNA regulation and evolved H_2 _in the light and consumed endogenous substrates.

## Conclusions

We have performed a deep-sequencing analysis of miRNAs in *Chlamydomonas reinhardtii *and provided a genome-wide, quantitative view of how sulfur-deprivation impacts the expression of small RNAs in *Chlamydomonas reinhardtii*. Our data confirm 24 known miRNAs and 23 predicted miRNAs with altered expression under sulfur-deprivation, most of which were up-regulated and 13 were -S library specific. Target predictions revealed that a variety of metabolic processes may be affected by changing the expression of miRNAs. Our study has delivered new insights into the role of miRNAs involved in sulfur-deprivation and provided a new approach to understand the biohydrogen production from the small RNA level in *Chlamydomonas reinhardtii*.

## Methods

### Growth of the algae

*Chlamydomonas reinhardtii *CC849 were obtained from *Chlamydomonas *Genetic Centre (c/o Dr. Elizabeth H. Harris, Department of Botany, Duke University, Durham, NC27706, USA). The algal strain was grown in a Tris-Acetate-Phosphate (TAP) medium at 25°C and under continuous cool-white fluorescent lamps(≈200 μmol photons m^-2 ^s^-1^). To impose S deprivation [6,8], the liquid cultures were grown into mid-logarithmic phase, algal cells were collected by centrifugation, were washed twice with liquid TAP medium without S (TAP-S, for 1 L of Medium: 2X Filner's Beijernicks Solution 25 ml; 1 M Potassium Phosphate 1 ml; Trace mineral solution 1 ml; Tris-Base 2.42 g; adjust pH to 7.0 by Glacial Acetic Acid. Sulfur-deprivation media (TAP-S) were prepared by replacement of the S-salts by their chloride counterparts). Equal numbers of cells were resuspended in TAP or TAP-S under continuous illumination for up to 72 h, with cell aliquots were collected for RNA isolation, and the sulfate concentration in the supernatant was determined by Dionex ICS-1100 ion chromatogram. Small RNA library construction was carried out as follows: for the + S library, RNA was isolated from the algal cells which were resuspended in sulfur-replete TAP media; for the -S library, RNA was isolated from the algal cells which were resuspended in sulfur-free media TAP-S.

### Preparation of total RNA

Total RNA was extracted using Trizol reagent (Invitrogen). The 72 h cells cultured at 25°C in TAP and TAP-S were collected. Total RNA was extracted according to the manufacturer's protocol. The quality of RNA was examined by using an Agilent 2100 Bioanalyzer. The same amount of total RNA was used to construct the two libraries and the samples were prepared in a similar manner. Sequencing of the two libraries was performed on the Illumina's Solexa Sequencer and the samples were run side by side.

### Small RNA library construction and high-throughput sequencing

After PAGE purification of small RNA molecules under 30 bases and ligation of a pair of Solexa adaptors to their 5'and 3'ends (Illumina, San Diego, CA. USA), the small RNA molecules were amplified using the adaptor primers for 17 cycles and the fragments around 90 bp (small RNA + adaptors) were isolated with agarose gel. The purified DNA was used directly for cluster generation and sequencing analysis using the Illumina's Solexa Sequencer according to the manufacturer's instructions. The image files generated by the sequencer were processed to produce digital-quality data. The following procedures were performed with Solexa to summarize the data: evaluation of the sequencing quality, calculation of the length distribution of small RNA reads, and filtration of the reads contaminated by rRNA, tRNA, mRNA, snRNA, and snoRNA. Finally, clean reads were compared with a miRBase database (release 15.0).

### Bioinformatics analysis

#### Data quality and length distribution

We eliminated some contaminant reads from the fq file and to obtain the final clean reads and then summarized the length distribution of these clean reads. Normally, length of small RNA is between 18 nt and 30 nt. The length distribution analysis was helpful to see the composition of small RNA samples. For example, miRNA is normally 21 nt or 22 nt, siRNA is 24 nt, and piRNA is 30 nt. The data was processed by the following steps: 1) Elimination of low quality reads (the criteria for this was listed in the explanation of meaning of each row in the result tables); 2) Elimination of reads with 5' primer contaminants; 3) Elimination of reads without 3' primer; 4) Elimination of reads without the insert tag; 5) Elimination of reads with poly A; 6) Elimination of reads shorter than 18 nt; 7) Summarization of the length distribution of the clean reads.

#### Mapping to genome

We mapped the small RNA tags to the genome by SOAP to analyze their expression and distribution on the genome. Program and Parameters: soap -v 0 -r 2 -s 7 -p 7 -a clean.fa -d ref_genome.fa -o match_genome.soap.

#### Summary of known miRNA alignment

We aligned the small RNA to the miRNA precursor of corresponding species (using mature miRNA if there was no precursor information of that species in miRBase14.0) to obtain the miRNA count as well as base bias on the first position of identified miRNAs with certain lengths and on each position of all identified miRNAs respectively.

#### siRNA identification

Small interfering RNA (siRNA) is a 22-24 nt long double-strand RNA, each strand of which is 2 nt longer than the other on the 3' end. According to this structural feature, we aligned tags from clean reads to each other to find sRNAs meeting this criteria. These tags might be potential siRNA candidates. Program and Parameters: Software developed by BGI-tag2siRNA.

#### Alignment to Genbank

We annotated the small RNA tags with rRNA, scRNA, snoRNA, snRNA and tRNA from Genbank and elimination of matched tags from unannotated tags. Program and Parameters: blastall -p blastn -FF -e 0.01

#### Alignment to Rfam

Annotation of the small RNA tags with sequences from Rfam and elimination of matched tags from unannotated tags. Program and Parameters: blastall -p blastn -FF -e 0.01

#### Exon and intron alignment

We aligned small RNA tags to exons and introns of mRNA http://genome.jgi-psf.org/Chlre3/Chlre3.download.ftp.html to find the degraded fragments of mRNA in the small RNA tags. Program and Parameters: Software developed by BGI-overlap

#### Small RNA annotation

We Summarized all prior alignments and annotation before. In the previous alignment and annotation, some small RNA tags may be mapped to more than one category. To make every unique small RNA map to only one annotation, we followed the following priority rule: rRNAetc(in which Genbank > Rfam) > known miRNA > repeat > exon > intron. Program and Parameters: Software developed by BGI-tag2annotation

#### Known miRNA expression profile

We aligned small RNA tags to the miRNA precursor/mature miRNA of corresponding species in miRBase14.0. To show detailed information of alignment, including structure of known miRNA precursor, length and count of tags from the sample, etc, click the miRNA id in the left table to see detailed information of that miRNA. Note: Only part of the known miRNA alignment are shown in this report.

#### Novel miRNA prediction

The characteristic hairpin structure of miRNA precursor was used to predict novel miRNA. We used the predictive software Mireap to predict novel miRNA by exploring the secondary structure, and the minimum free energy of the unannotated small RNA tags which could be mapped to genome. Mireap can be accessed from the following link: http://sourceforge.net/projects/mireap/

#### Program and parameters

Software developed by BGI- Mireap Minimal miRNA sequence length (18); Maximal miRNA sequence length (25); Minimal miRNA reference sequence length (20); Maximal miRNA reference sequence length (23); Maximal copy number of miRNAs on reference (20); Maximal free energy allowed for a miRNA precursor (-18 kcal/mol); Maximal space between miRNA and miRNA* (300); Minimal base pairs of miRNA and miRNA* (16); Maximal bulge of miRNA and miRNA* (4); Maximal asymmetry of miRNA/miRNA* duplex (4); Flank sequence length of miRNA precursor (20); ram and Parameters: blastall -p blastn -FF -e 0.01.

### Differential expression of known miRNA

Comparison of the known miRNA expression between two samples to determine the differentially expressed miRNA. The procedures are shown as below: (1) Normalize the expression of miRNA in two samples (control and treatment) to get the expression of transcript per million(TPM). Normalization forum:Normalized expression = Actual miRNA count/Total count of clean reads*1000000; (2) Calculate fold-change and P-value from the normalized expression according the Bayesian method developed by Audic and Claverie (1997) [28]. Then generate the log2 ratio plot and scatter plot.

Fold-change forum: Fold change = log 2 (treatment/control)

P-value forum:

$$p\left(x|y\right)=\left(\frac{N_{2}}{N_{1}}\right)\frac{\left(x+y\right)!}{x!y!{\left(1+\frac{N_{2}}{N_{1}}\right)}^{\left(x+y+1\right)}}\begin{array}{c} C\left(y\leq y_{\min}|x\right)=\overset{y\leq y_{\min}}{\underset{y=0}{\sum }}p\left(y|x\right)\\ D\left(y\geq y_{\max}|x\right)=\overset{\infty }{\underset{y\geq y_{\max}}{\sum }}p\left(y|x\right) \end{array}$$

The N1 and x represent total counts of clean reads and normalized expression level of a given miRNA in sRNA library of + S sample, respectively. The N2 and y represent total counts of clean reads and normalized expression levels of a given miRNA in sRNA library of -S sample, respectively.

### Quantitative RT-PCR validation of miRNAs

Sulfur-deprivation procedure is the same as previously described. Both total RNA from 72 h cells cultivated in TAP and TAP-S were isolated respectively for quantitative RT-PCR using the TRIZOL reagent (Invitrogen, Life Technologies, Carlsbad, CA). Low molecular weight RNA and high molecular weight RNA were separated with 4 M LiCl. sRNAs were polyadenylated at 37°C for 60 min in a 50 μl reaction mixture with 1.5 μg of total RNA, 1 mM ATP, 2.5 mM MgCl2, and 4 U poly(A) polymerase (Takara, Japan). Poly (A)-tailed sRNA was recovered by phenol/chloroform extraction and ethanol precipitation. The sRNAs were dissolved, treated with RNase-free DnaseI (Takara, Japan) and reversely transcribed using poly (T) adapter. Real-time PCR was performed using SYBRR Green Real-time PCR Master Mix (Toyobo, Osaka, Japan) and all the primers used were as listed in Additional file 2: Table S1. For each reaction, 1 μL of diluted cDNA (equivalent to 100 pg of total RNA) was mixed with 10 μL of 2 × SYBR green reaction mix (SYBRR Green qRT-PCR Master Mix; Takara, Japan), and 5 pmol of the forward and the reverse primers were added to make a final volume of 20 μL. The conditions for the PCR amplification were as follows: polymerase activation was conducted at 95°C for 30 s; followed by 40 cycles at 95°C for 5 s, 60°C for 31 s. The specificity of the primer amplicons was tested by analysis of a melting curve. The U4 snoRNA was used as a reference gene in the real-time PCR detection of miRNAs. The data was analyzed using the 2^--ΔΔCt ^program, all with an R^2 ^above 0.998. For this quantitative RT-PCR analysis, 3 technical replicates and 2 biological replicates were used.

## Competing interests

The authors declare that they have no competing interests.

## Authors' contributions

LS contributed to execute experiments and write the manuscript, ZH contributed to design the research plans and write the manuscript. Both authors read and approved the final manuscript.

## Supplementary Material

Additional file 1 **Figure S1 Mapping of small RNAs in the + S (a) and -S (b) libraries to genome by SOAP**. Y axis represents the number of small RNA tags that locate on each chromosome. The numbers of sRNAs on the sense strand of chromosome are positive (shown in blue), and those on the antisence strand of chromosome are negative (shown in red). X axis shows the chromosomes. Click here for file

Additional file 2 **Table S1 The primers used in this experiment**. Click here for file
