# Supplementary figures and images for: Characterization and differential expression of microRNAs elicited by sulfur deprivation in Chlamydomonas reinhardtii
Source: BMC Genomics. 2012 Mar 22;13:108. doi: 10.1186/1471-2164-13-108 (PMC3441669; doi:10.1186/1471-2164-13-108)

## Slide 1
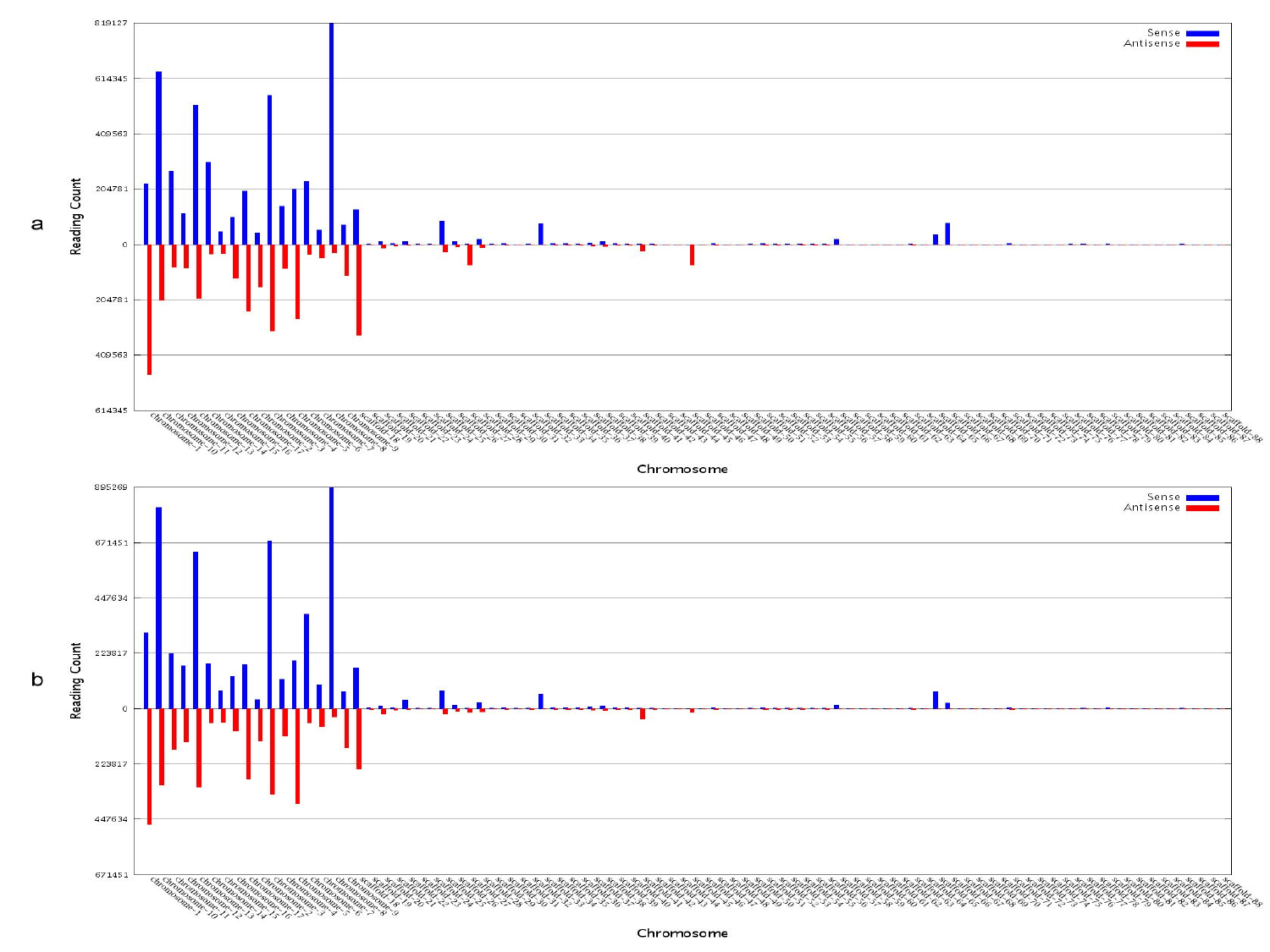

Supplement: Additional file 1 — Figure S1 Mapping of small RNAs in the + S (a) and -S (b) libraries to genome by SOAP. Y axis represents the number of small RNA tags that locate on each chromosome. The numbers of sRNAs on the sense strand of chromosome are positive (shown in blue), and those on the antisence strand of chromosome are negative (shown in red). X axis shows the chromosomes. [file 1471-2164-13-108-S1.PPT]
