# Supplementary material for: Characterization and differential expression of microRNAs elicited by sulfur deprivation in Chlamydomonas reinhardtii
Source: BMC Genomics. 2012 Mar 22;13:108. doi: 10.1186/1471-2164-13-108 (PMC3441669; doi:10.1186/1471-2164-13-108)
Supplement: Additional file 2 — Table S1 The primers used in this experiment. [file 1471-2164-13-108-S2.DOC]

**Table S1 The primers used in this experiment**

| **Title** | **Primer** | **seqence** |
| --- | --- | --- |
| Poly T Adapter | Stem-loop reverse primer | GCGAGCACAGAATTAATACGACTCACTATAGGTTTTTTTTTTTTVN |
| Universal R | Universal reverse primer | GCGAGCACAGAATTAATACGACTCAC |
| miR906-3p-F | Forward primer | TCCGATAAAGCTTCCCCCTGC |
| miR906-5p-F | Forward primer | CGGTTGGTGGGCGTGATCAGC |
| miR909.3-F | Forward primer | TTCAGGGTCAAGTTTGCATGC |
| miR913-F | Forward primer | TGCACACTTGCGAGTCCGTGG |
| miR917-F | Forward primer | TTTCACGGTTATGTTCGAAG |
| miR1145.1-F | Forward primer | TTGGGGCCCAGCAGGTCCTGG |
| miR1145.2-F | Forward primer | TGGCGTTGACCCTGTCGGTGG |
| miR1156.2-F | Forward primer | TTCAGCTGGAGCTTCAGGCAC |
| miR1158-F | Forward primer | ACTTGGAGGAGGCCACTGGC |
| miR1146-F | Forward primer | ATGGGTCCGATCGGGAAGCT |
| miR1150.3-F | Forward primer | TGCAGCGGCGACTGGGGCCGA |
| miR1159.1-F | Forward primer | TGCCACAGTGCCCGATTGCCG |
| n30 | Forward primer | TCAAAGCTAGGAGCCATGAAG |
| n62 | Forward primer | TGACATGCGGTGAATGTGAAT |
| U4-F | Forward primer | CAAAAGGCCCGACAGAAAT |
| U4-R | Reverse primer | GTGAGGTCTAACCGAGTCGC |
